# A wearable hip-assist robot reduces the cardiopulmonary metabolic energy expenditure during stair ascent in elderly adults: a pilot cross-sectional study

**DOI:** 10.1186/s12877-018-0921-1

**Published:** 2018-09-29

**Authors:** Dong-Seok Kim, Hwang-Jae Lee, Su-Hyun Lee, Won Hyuk Chang, Junwon Jang, Byung-Ok Choi, Gyu-Ha Ryu, Yun-Hee Kim

**Affiliations:** 1 Department of Physical and Rehabilitation Medicine, Center for Prevention & Rehabilitation, Heart Vascular Stroke Institute, Samsung Medical Center, Sungkyunkwan University School of Medicine, Irwon-ro 81, Gangnam-gu, Seoul, 06351 Republic of Korea; 2 Department of Health Sciences and Technology, SAIHST, Sungkyunkwan University, Irwon-ro 81, Gangnam-gu, Seoul, 06351 Republic of Korea; 3 Samsung Advanced Institute of Technology, Samsung Electronics, 130 Samsung-ro, Yeongtong-gu, Suwon-si, Gyeonggi-do 16678 Republic of Korea; 4 Department of Neurology, Neuroscience Center, Samsung Medical Center, Sungkyunkwan University School of Medicine, Irwon-ro 81, Gangnam-gu, Seoul, 06351 Republic of Korea; 5 Office of Biomechanical science, Research Center for Future Medicine, Samsung Medical Center, Sungkyunkwan University, Irwon-ro 81, Gangnam-gu, Seoul, 06351 Republic of Korea

**Keywords:** Wearable hip assist robot, Elderly adult, Metabolic energy expenditure, Stair ascent

## Abstract

**Background:**

Stair ascent is one of the most important and challenging activities of daily living to maintain mobility and independence in elderly adults. Recently, various types of wearable walking assist robots have been developed to improve gait function and metabolic efficiency for elderly adults. Several studies have shown that walking assist robots can improve cardiopulmonary metabolic efficiency during level walking in elderly. However, there is limited evidence demonstrating the effect of walking assist robots on cardiopulmonary metabolic efficiency during stair walking in elderly adults. Therefore, the aim of this study was to investigate the assistance effect of a newly developed wearable hip assist robot on cardiopulmonary metabolic efficiency during stair ascent in elderly adults.

**Methods:**

Fifteen healthy elderly adults participated. The Gait Enhancing Mechatronic System (GEMS), developed by Samsung Electronics Co., Ltd., Korea, was used in the present study. The metabolic energy expenditure was measured using a K4b^2^ while participants performed randomly assigned two conditions consecutively: free ascending stairs without the GEMS or robot-assisted ascending stair with the GEMS.

**Results:**

There were significant differences in the oxygen consumption per unit mass (ml/min/kg), metabolic power per unit mass (W/kg) and metabolic equivalents (METs) values between the GEMS and NoGEMS conditions. A statistically significant difference was found between the two conditions in net oxygen consumption and net metabolic power, with a reduction of 8.59% and 10.16% respectively in GEMS condition (*p* < 0.05). The gross oxygen consumption while climbing stairs under the GEMS and NoGEMS conditions was equivalent to 6.38 METs and 6.85 METs, respectively.

**Conclusion:**

This study demonstrated that the GEMS was helpful for reducing cardiopulmonary metabolic energy expenditure during stair climbing in elderly adults. The use of the GEMS allows elderly adults to climb stairs with less metabolic energy, therefore, they may experience more endurance in stair climbing while using the GEMS.

**Trial registration:**

NCT03389165, Registered 26 December 2017 - retrospectively registered

## Background

Stair walking (ascending and descending stairs) is one of the most important and challenging activities of daily living required to maintain mobility and independence [1]. However, stair walking demands a variety of physical functions such as proper lower limb strength, joint range of motion, kinesthetic intelligence, and visual processing for safe and coordinated locomotion [2, 3]. Furthermore, stair ascent increases the metabolic energy expenditure compared with level walking [4]. According to the metabolic equivalents (METs) that are a physiological measure expressing the energy expenditure of physical activities and are defined as the ratio of metabolic rate during a specific physical activity to resting metabolic rate, normative walking requires 3.3 METs, whereas normative stair ascent and stair ascent while carrying a moderate load require 5 and 8 METs, respectively [5]. Especially for elderly adults who expend an average of 20% more metabolic energy during walking than young adults [6], stair walking is one of the tasks that places a large burden on daily life. The higher metabolic energy expenditure increases the sense of task effort and fatigue and therefore can contribute to increased risk of falls in elderly adults [7]. For the elderly, a reduction in the energy expenditure of level and stair walking is important elements in preventing falls and maintaining quality of life. Hence, in order to improve cardiopulmonary metabolic efficiency of elderly adults, it is essential to develop useful methods such as physical training utilizing resistance and aerobic exercise, balance training, and assistance using robotic devices.

Specifically, various kinds of wearable-type walking assist robots that provide assistance torque around the hip, knee, and ankle joints are being developed. Recently, many studies using walking assist robots have been performed to identify changes in gait function in elderly adults [8–12]. Several positive effects of walking assist robots on gait function such as gait speed and walking parameters in the elderly have been reported by many research companies and institutions [8–10]. A well-known walking assist robot, the Stride Management Assist Device (SMA, Honda R&D Corporation, Japan), improved gait parameters and reduced glucose metabolism in the lower limb muscles in elderly adults [8]. Shimada et al. reported that a 3-month walking intervention program using the SMA device was useful in improving gait function and the efficiency of muscle efforts during walking in elderly adults [9]. In particular, previous studies have examined the effects of robotic assist on metabolic measurements such as oxygen consumption per unit mass, metabolic power per unit mass, and metabolic equivalents. Several studies have also shown that walking assist robots were effective in reducing metabolic energy expenditure during walking in elderly adults [10–12]. Jin et al. reported that a new soft robotic suit reduced net metabolic power by an average of 5.9% in the powered-on condition compared with the powered-off condition in nine elderly adults during walking [11]. Galle et al. reported that bilateral ankle-foot exoskeletons could reduce energy expenditure by 12% in comparison with the powered-off condition in elderly adults. This device also resulted in a 4% reduction in energy expenditure compared to walking in normal shoes [12]. Therefore, metabolic measurement is a very important variable for verifying the assist effect of robotic devices.

Recently, Samsung Advanced Institute of Technology developed the Gait Enhancing Mechatronic System (GEMS, Samsung Electronics Co., Ltd., Korea), which aids the hip joint flexion and extension movement of the wearer during walking. In a previous study, elderly people demonstrated improved gait function, decreased muscle effort, and reduced energy expenditure during walking with the GEMS compared to walking without the GEMS [10, 13].

Most of the studies using walking assist robots for the elderly have been conducted to observe the effect of the robots on gait function and metabolic efficiency in level walking. However, there is limited evidence demonstrating the effect of walking assist robots on gait function and metabolic efficiency in stair walking in elderly adults. Only a few studies on walking assist robots for stair walking have been reported in the literature [14, 15].

Therefore, the aim of this pilot cross-sectional study was to identify the assistance effect of the GEMS as a newly developed wearable hip-assist robot during stair ascent by comparing the energy expenditure of elderly adults with and without the GEMS. We hypothesized that using the GEMS during stair ascent would reduce energy expenditure and improve stair climbing speed compared to stair ascent without GEMS.

## Methods

### Participants

We recruited elderly adults who met the inclusion and exclusion criteria. Fifteen healthy elderly adults participated in the present study [16]. Table 1 shows the characteristics of the participants. Inclusion criteria of this study were as follows: 1) absence of a history of musculoskeletal or central nervous system diseases and 2) physical performance abilities with short physical performance battery scores of 8 or higher (mild and minimal limitations range without any problems in activity of daily living) [17]. Exclusion criteria were as follows: 1) absence of the ability to walk independently due to visual field defects, fractures, or severe muscle weakness, 2) severe dizziness that might lead to falls, and 3) cognitive disorders that might be difficult to understand accurately in this study. All participants provided informed consent before participating in the study. This study protocol was approved by the Samsung Medical Center Institutional Review Board.

**Table 1:** Characteristics of the participants

Characteristic	Value
Sex (male / female)	6 / 9
Age, years (mean ± SD)	74.33 ± 4.56
Height, cm (mean ± SD)	159.40 ± 5.73
Weight, kg (mean ± SD)	59.80 ± 9.24
BMI (mean ± SD)	23.54 ± 3.39
SPPB, points (mean ± SD)	10.53 ± 1.36

*SD* Standard Deviation, *BMI* Body Mass Index, *SPPB* Short Physical Performance Battery

### Wearable hip-assist robot

**Fig. 1:**
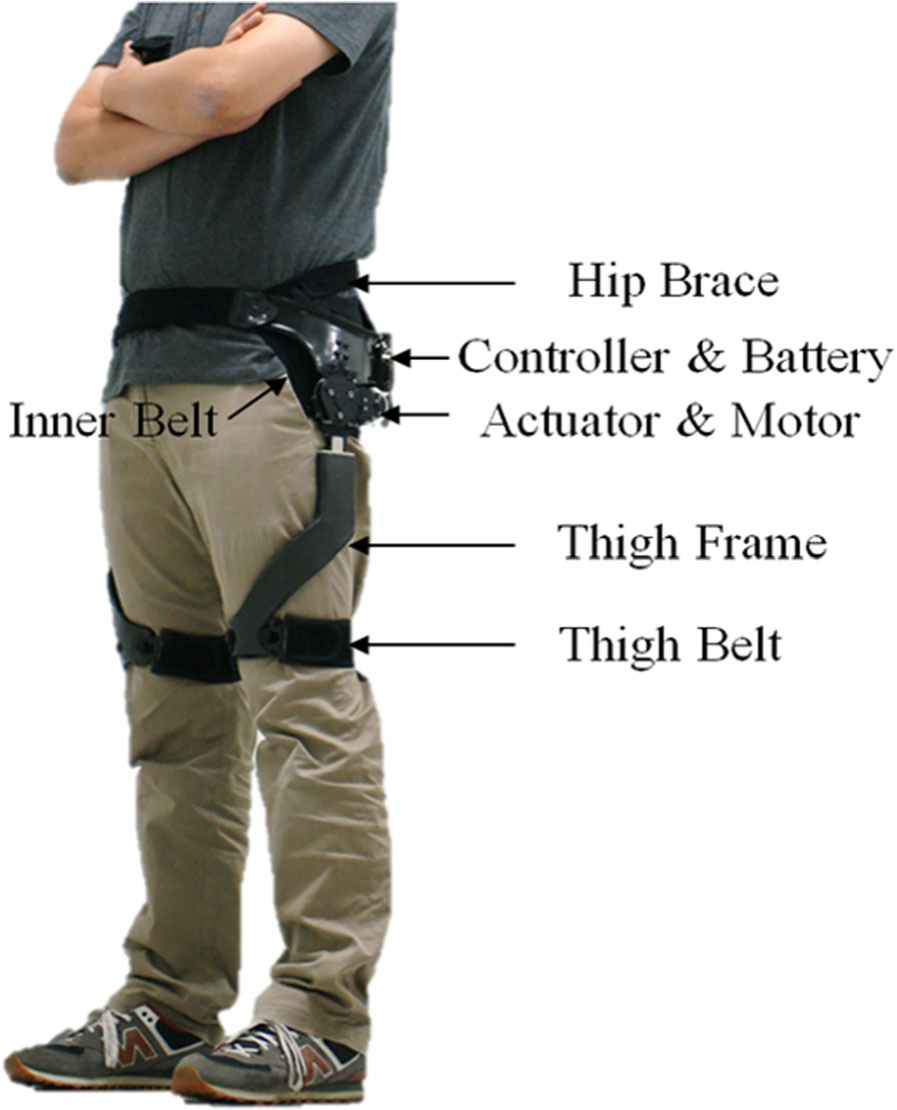
Gait Enhancing Mechatronic System (GEMS)

As show in Fig. 1, the GEMS worn around the waist is composed of a pair of actuators that generate assistance power to the left and right hip joints, a hip brace around the waist, a pair of thigh frames for transmission of assistance torque from the actuators to the thighs, and fabric belts at the ends of the thigh frames. The GEMS is available in two sizes, small (for hip circumferences 70~ 90 cm) and medium (for hip circumferences 90~ 100 cm), and each size can be adjusted according to the body size of the individual. Two 70-W brushless DC motors positioned around the hip joints generate assistance torque and deliver the generated torque to each hip joint with a maximum torque of 12 Nm through a 75:1 multi-stage gear system. Each joint has one active degree of freedom for flexion and extension in the sagittal plane by electric actuators and one passive hinge below the active degree of freedom for abduction and adduction in the frontal plane. The range of motion is 120/45 degrees for flexion/extension and 20/20 degrees for abduction/adduction. The battery, CPU, inertial measurement unit (IMU) sensor, and motor driving units are all located on the back of the device. An IMU sensor is used to calculate gait state from the hip joint angles and to determine the assistance torque pattern. The GEMS can work continuously for 1.3 h under normal operating conditions, and its total weight is 2.8 kg.

**Fig. 2:**
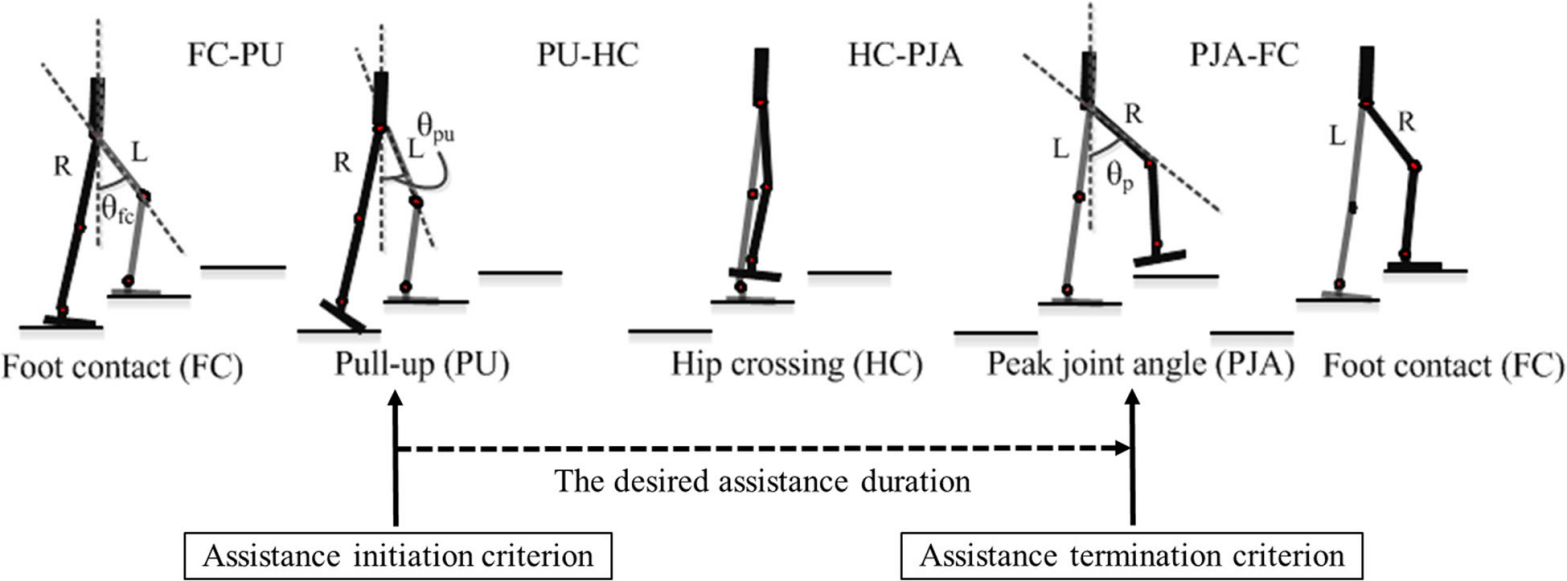
Phases of the gait cycle divided into 4 as follows: foot contact to pull-up (FC-PU), pull-up to hip crossing (PU-HC), hip crossing to peak joint angle of a swing leg (HC-PJA), and peak joint angle of a swing leg to foot contact (PJA-FC), and 2 events (PU and PJA) used as initiation and termination criterion of assistance torque for the desired assistance duration, respectively. FC = Foot Contact, PU = Pull Up, HC = Hip Crossing, PJA = Peak Joint Angle

The assistance strategy of the GEMS for stair ascent utilizes a foot contact event estimated by IMU sensor data and detects user intention as well as reflect user preference [18]. In this strategy, a gait cycle for stair ascent is divided into 4 phases to detect user intention more easily and exactly in real time. The divided gait cycle is as follows: foot contact to pull-up (FC-PU), pull-up to hip crossing (PU-HC), hip crossing to peak joint angle of a swing leg (HC-PJA), and peak joint angle of a swing leg to foot contact (PJA-FC) (Fig. 2). The moments of each events are determined by hip joint angles estimated by two joint angle sensors. Also, this assistance strategy applies criteria for the initiation and termination of assistance torque by recognizing user intention. Pull-up, which is an obvious clue indicating user intention to climb stair, is used as assistance initiation criterion during stair ascent. At the moment of pull-up event detection, the desired assistance durations are determined, and the assistance torques for each stance and swing leg are then generated for a new step. The generated assistance torques are applied to each stance and swing leg in a feedforward manner for the desired assistance duration until the assistance termination criterion is met and are finished at the peak joint angle exploited as assistance termination criterion (Fig. 2). In addition, at the last minute of the peak joint angle event, decay flexion and extension torque profiles are applied to terminate the assistance torque profiles rapidly and smoothly.

### Experimental protocol

The stair ascent trials were conducted at the Samsung Medical Center, Seoul, Korea. Metabolic energy expenditure was measured while participants climb stairs from the first basement level to the fourth floor, a total of 16 flights comprised of 128 steps with each step at 17 cm in height, for a total vertical displacement of 21.76 m.

Prior to initiation of the trials, the physical performance abilities of participants were evaluated through a short physical performance battery test to make sure that they meet the inclusion criteria of scores 8 or higher, and basic physical information (age, gender, height, body weight, waist measurement, leg length, and blood pressure), disease information, and pain information were recorded. In order for participants to wear the GEMS comfortably, the size of the GEMS was adjusted by a researcher. In order to adapt participants to the GEMS and familiarize them with stair ascent with the GEMS, all participants ascended and descended a 6-step custom-made staircase installed in the laboratory for 10 min. While all participants performed stair walking, the assist torque of the GEMS was determined to be the most comfortable level for the individual.

The K4b^2^ system (Cosmed, Rome, Italy) based on true breath-by-breath technology was used to measure metabolic energy expenditure during stair ascent with and without the GEMS. Before the stair ascent test, all participants were equipped with a K4b^2^ analyzer unit that was strapped to the chest, and a facial mask connected to an analyzer unit was placed on the nose and mouth. Also, a heart rate monitor (T31 transmitter, POLAR, USA) was strapped around the participant’s chest to measure heart rate. Participants were instructed not to talk during the trials.

The stair ascent trials were performed under two different conditions: free ascent without the GEMS (NoGEMS) and robot-assist ascent with the GEMS (GEMS). The experimental protocol of the present study was as follows. All participants stood for 5 min to obtain baseline variables. Participants were then asked to climb stairs from the first basement level to the fourth floor in a step-over-step manner under two different conditions, GEMS and NoGEMS. The GEMS and NoGEMS conditions were conducted in a random order, and just before the second condition, participants stood for 2~ 3 min to obtain baseline variables once more. Participants rested between the two conditions for 10 min to restore metabolic rate to baseline level. During stair climbing, time was recorded with a stopwatch to compare the difference in climbing speed between the two conditions.

### Measurement tool

The K4b^2^ system, which was worn in a harness on the shoulders, was used to measure metabolic energy expenditure during stair ascent with and without the GEMS. The K4b^2^ system is a computerized portable system for cardiopulmonary gas exchange measurements based on true breath-by-breath analysis and allows the user to measure the physiological response to exercise without limitations. This device is composed of a facial mask worn over the participant’s nose and mouth, a gas sample line, an analyzer unit strapped to the participant’s chest, and a battery-operated unit. It is designed to measure the parameters of oxygen consumption, carbon dioxide emission, and ventilation with several sensors. The K4b^2^ analyzer unit was calibrated before each test for accurate sensor operation. Calibrations of the flow turbine and gas analyzers were performed using a 3-l syringe and gas mixtures, respectively. Heart rate was measured using a heart rate monitor strapped around the participant’s chest, and heart rate data were transmitted to a portable device.

### Data analysis

Metabolic energy expenditure during stair ascent was obtained by the K4b^2^ system. To estimate the metabolic energy expenditure, we applied net oxygen consumption (ml/min/kg) and net metabolic power (W/kg) computed from VO_2_ (L/min) and the respiratory exchange ratio (RER) recorded by the K4b^2^ system because net values give a more direct indication of metabolic efficiency than gross values [19]. The VO_2_ and RER were computed as the mean values for the last minute of each condition to compare the metabolic energy expenditure consumed for the same time in climbing stairs and were used to calculate the energy cost (kcal/min) of each condition using the regression equation reported by Zuntz [20]. The regression equation which is based on the thermal equivalent of VO_2_ for non-protein respiratory equivalent was used to calculate the energy cost through mean values of the VO_2_ and RER for each condition.

$$ \mathrm{Energy}\ \mathrm{cost}={\mathrm{VO}}_2\times \left(1.2341\times \mathrm{RER}+3.8124\right) $$

Then, energy cost was used to calculate metabolic power of each condition in watts through the conversion factor 69.78 W·kcal/min as follows:

$$ \mathrm{Metabolic}\ \mathrm{power}=\mathrm{Energy}\ \mathrm{cost}\kern0.5em \times 69.78 $$

After that, in order to obtain the net metabolic power of each condition, we subtracted the resting metabolic power form each gross metabolic power [21]. The net metabolic power of each condition was normalized by dividing by body weight (W/kg) and compared with average values.

The average values of the METs, which are a physiological measure expressing the energy expenditure of physical activities, were computed as the average values for the last minute of the GEMS and NoGEMS conditions. The METs intensity during stair ascent with and without the GEMS was calculated by dividing average oxygen consumption (ml/min/kg) by 3.5 ml/min/kg.

### Statistical analysis

Paired t-tests were used to compare significant differences in oxygen consumption, metabolic power, METs values, and stair climbing cadence between the GEMS condition and the NoGEMS condition. Statistical analysis was conducted with Statistical Package for the Social Sciences ver. 22.0 for Windows software (SPSS Inc., Chicago, IL, USA). The statistical significance level for the paired t-tests was set at *p* < 0.05.

## Results

**Fig. 3:**
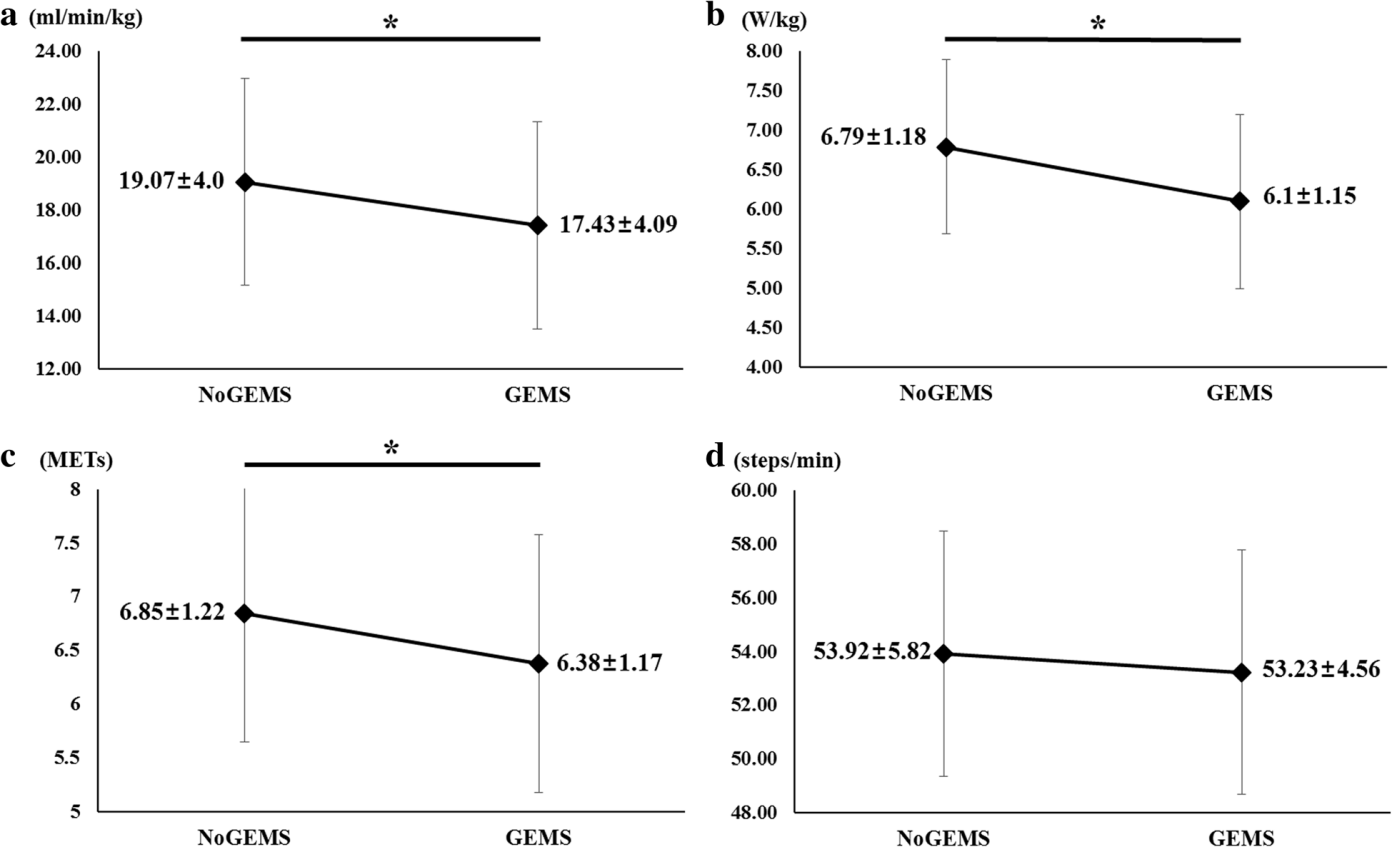
**a**) Comparison of average net oxygen consumption per unit mass (ml/min/kg) under the NoGEMS and GEMS conditions (* *p* < 0.05). **b**) Comparison of average net metabolic power per unit mass (W/kg) under the NoGEMS and GEMS conditions (* *p* < 0.05). **c**) Comparison of average metabolic equivalents (METs) values under the NoGEMS and GEMS conditions (* *p* < 0.05). **d**) Comparison of average stair climbing cadence (steps/min) under the NoGEMS and GEMS conditions (*p* = 0.388)

As show in Fig. 3a), we found significant differences between net oxygen consumption per unit mass in the two different conditions. The mean of net oxygen consumption per unit mass with and without the GEMS was 17.43 ± 4.09 and 19.07 ± 4.00 ml/min/kg, respectively. In addition, the mean of resting oxygen consumption per unit mass obtained before first and second conditions was 4.64 ± 0.64 and 5.11 ± 0.84 ml/min/kg, respectively. A statistically significant difference was found between the GEMS and NoGEMS conditions in net oxygen consumption per unit mass, with a reduction of 8.59%, corresponding to 1.64 ml/min/kg under the GEMS condition (*p* = 0.013).

Figure 3b) shows that stair ascent with the GEMS required an average of 6.10 ± 1.15 W/kg, while stair ascent without the GEMS required an average of 6.79 ± 1.18 W/kg of net metabolic power per unit mass. This difference was statistically significant, with a reduction of 10.16%, corresponding to 0.69 W/kg under the GEMS condition (*p* = 0.001). The mean of resting metabolic power per unit mass obtained before first and second conditions was 1.59 ± 0.17 and 1.67 ± 0.24 W/kg, respectively.

In addition, we calculated the METs to compare the intensity of climbing 128 steps with and without the GEMS. The gross oxygen consumption of climbing stairs under the GEMS and NoGEMS conditions was equivalent to 6.38 METs and 6.85 METs, respectively (Fig. 3c). There was a statistically significant difference in METs value between the two conditions, with a difference of 0.47 METs (*p* < 0.05). Figure 3d) shows the average stair climbing cadence (steps/min) under the GEMS and NoGEMS conditions. The stair climbing with GEMS took an average of 145 s and cadence was an average of 53.25 ± 4.56 steps/min. While the stair climbing without GEMS took an average of 144 s and cadence was an average of 53.92 ± 5.82 steps/min. There was no significant difference in stair climbing cadence between the two conditions (*p* = 0.388).

In summary, oxygen consumption per unit mass and metabolic power per unit mass were significantly reduced during stair ascent with the GEMS compared to free gait without the GEMS.

## Discussion

The aim of this study was to identify the assistance effect of the GEMS by comparing the energy expenditure between the GEMS and NoGEMS conditions during stair ascent in elderly adults. In the present study, the GEMS was found to reduce oxygen consumption and metabolic power during stair ascent in elderly adults. Furthermore, we demonstrated the assistance effect of the GEMS on stair climbing based on the observation of a significant reduction in energy expenditure in elderly adults with the GEMS.

The gait of the elderly is typically characterized by short step length, slow walking velocity, reduced range of joint motion, and reduced balance [7]. These characteristics increase the risk of falls and unnecessary walking energy expenditure [22]. Metabolic energy expenditure is an important indicator of physical capability because it represents an individual’s physiological ability to supply energy to support various physical activities [23]. Previous studies have reported that elderly adults consume 20% more cardiopulmonary metabolic energy than young adults during walking [24, 25]. Furthermore, elderly adults were dependents on the higher metabolic energy expenditure during stair ascent than level walking [26]. Several studies have suggested that degradation in physical function in the elderly, such as decreased sense of balance and loss of muscle strength, might be related to increase in metabolic energy expenditure [24, 27]. Waters et al. reported that elderly with restrictions in range of motion in the lower limb joints expended more metabolic energy expenditure during walking than adults without such restrictions [28]. Also, several studies reported that decline in step length and gait speed contributed to increase in metabolic energy expenditure in elderly adults [24, 29]. As reported several studies, the increase in metabolic energy expenditure in elderly adults is related to gait function. In a previous study utilizing the GEMS, Lee et al. reported the effects of the GEMS on gait speed, cadence, stride length, step width, and muscle effort during level walking and demonstrated that the GEMS improved gait function and cardiopulmonary metabolic efficiency, with a reduction of approximately 7% in oxygen consumption in elderly adults [10]. The result of our study found that the net metabolic energy expenditure reduction was about 10.2% in the GEMS compared to NoGEMS condition. In the other hand, a recent study using hip-assist soft exoskeleton reported reduction of net metabolic energy expenditure by 17.4% during treadmill gait in young adults [30]. Therefore, further studies are needed to directly compare the effect of different robotic devices on metabolic energy expenditure for diverse ADL conditions in elderly adults. Although the GEMS is about 2 kg heavier than the soft exoskeleton, it is meaningful result that GEMS reduces the metabolic energy expenditure by 10.16% during stair ascent in elderly adults.

Also, this study observed the metabolic power of climbing stairs to compare walking efficiency between assistive gait with the GEMS and free gait without the GEMS. The unit of metabolic power is W (watt) or J/s, indicating how efficiently an individual works per unit time. The calculated net metabolic power, which is based on the measured oxygen consumption (VO_2_) and carbon dioxide emission (VCO_2_) values, was 362.13 W under the GEMS condition and 402.93 W under the NoGEMS condition in this study. The present results indicate that the assistance of the GEMS during stair ascent influences walking efficiency, with a reduction of 10.16% in net metabolic power in the elderly. However, as presented in the results, there was no statistically significant difference in stair climbing speed between the GEMS and NoGEMS conditions. Namely, the use of the GEMS allowed elderly adults to perform the same tasks using less energy than without the GEMS. Thus, the GEMS aids elderly adults in using metabolic energy more efficiently during stair ascent. From these results, we suggest that the reduction in metabolic energy expenditure caused by the aid of the GEMS on stair climbing might closely relate to the reduction of muscles activation or improvement of gait function in elderly adults. Recently, various kinds of wearable-type walking assist robots that provide assistance torque around the hip, knee, and ankle joints have been developed for energy-efficient walking and improvement in gait function in elderly adults and patients with gait impairment. Previous studies have reported the effects of other walking assist devices on gait function and metabolic efficiency during walking. Jin et al. reported that a soft robotic suit, which provides an assistive force for hip flexion through winding belts, significantly reduced net metabolic power by an average of 5.9% and increased maximum hip angle by an average of 5.4% during walking in elderly adults [31]. Some energy expenditure effects of the wearable hip assist devices were reported even in young adults. Young et al. (2017) demonstrated that a pneumatic hip exoskeleton improved metabolic efficiency during walking, with a reduction of 9.7% in net metabolic power with the ideal timing of hip flexion [32]. Additionally, Ding et al. showed that soft exosuits, which provide assistance forces to the hip joints, reduced the metabolic power of walking by 4.6%, effectively improving walking efficiency [33]. There are many differences between these previous studies and the present study. The main difference is that previous studies examined level walking, while the present study was conducted while climbing stairs, which requires approximately 30–40% more cardiopulmonary metabolic energy compared with level walking [34, 35]. Also, previous studies compared net metabolic power between power-on and power-off conditions both with the walking assist device attached in order to reduce the advantage of free gait without the device load. The present study compared net metabolic power between stair climbing without the GEMS and stair climbing with the GEMS, which weighs 2.8 kg.

In addition, the METs were observed to compare the physical intensity of climbing 128 stairs with and without the GEMS. The METs are a physiological measure expressing the energy expenditure of physical activities and is defined as the ratio of metabolic rate during a specific physical activity to resting metabolic rate, which is an oxygen consumption at rest of approximately 3.5 ml/min/kg. As such, the physical activity at 3 METs demands three times a resting oxygen consumption of approximately 3.5 ml/min/kg. According to reports from the Compendium of Physical Activities, the METs values during stair climbing while carrying a load of approximately 3 kg and 9 kg were 5 and 6 METs, respectively. The METs values estimated by the Compendium of Physical Activities indicate that, as the load carried increases during stair climbing, the METs values increase. However, our measured results showed that the use of the GEMS during stair climbing decreased the METs values by approximately 0.5 METs, despite the increased device load of 2.8 kg. Therefore, the results from the present study indicate that use of the GEMS has a substantial benefit on reduction in metabolic energy expenditure during stair climbing in elderly adults.

This study has a limitation. Participants of our study had relatively good functional status and elderly people with severe gait disorder were not involved. For this sub-population, who have limitations in muscle power or postural stability for independent walking or stair climbing, further study is needed to assess the clinical effects of the GEMS on efficiency of walking or stair climbing.

## Conclusions

In this study, we found that the GEMS, which is a newly developed wearable hip assist robot designed to improve gait function and metabolic efficiency, is helpful for reducing cardiopulmonary metabolic energy expenditure during stair ascent in elderly adults. Although there was no significant difference in stair climbing speed between the GEMS and NoGEMS conditions, this study demonstrates that the use of the GEMS allows elderly adults to climb stairs with less cardiopulmonary metabolic energy expenditure compared to stair climbing without the GEMS. Therefore, elderly adults may experience more endurance in stair climbing while using the GEMS.

## Abbreviations

GEMS: Gait Enhancing Mechatronic System
IMU: Inertial measurement unit
METs: Metabolic equivalents
RER: Respiratory exchange ratio
